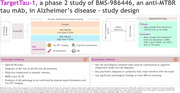# TargetTau‐1: Design of a phase 2 trial to evaluate the efficacy, safety, and tolerability of BMS‐986446, an anti‐MTBR tau monoclonal antibody, in patients with early Alzheimer’s disease

**DOI:** 10.1002/alz.094677

**Published:** 2025-01-09

**Authors:** Christopher H. van Dyck, Anja Kahl, Grigor Abelian, Mark Donovan, Manuj Ahuja, David Watson, Rik Ossenkoppele, Takeshi Iwatsubo, Oskar Hansson

**Affiliations:** ^1^ Alzheimer’s Disease Research Unit, Yale School of Medicine, New Haven, CT USA; ^2^ Bristol Myers Squibb, Princeton, NJ USA; ^3^ Alzheimer’s Research and Treatment Center, Wellington, FL USA; ^4^ Alzheimer Center Amsterdam, Neurology, Vrije Universiteit Amsterdam, Amsterdam UMC location VUmc, Amsterdam Netherlands; ^5^ Amsterdam Neuroscience, Neurodegeneration, Amsterdam Netherlands; ^6^ Clinical Memory Research Unit, Department of Clinical Sciences, Lund University, and Memory Clinic, Skåne University Hospital, Malmö Sweden; ^7^ Department of Neuropathology, Graduate School of Medicine, University of Tokyo, and the National Center of Neurology and Psychiatry, Tokyo Japan

## Abstract

**Background:**

Alzheimer’s disease (AD) is the leading cause of cognitive impairment and dementia with rising prevalence, morbidity, and mortality. Limited treatment options highlight a significant unmet need.

AD is characterized pathologically by extracellular accumulation of Aβ peptide‐containing plaques and intracellular neurofibrillary tangles containing aggregates of the microtubule‐associated protein tau, leading to neuronal and synaptic loss, neuroinflammation, and brain atrophy.

The microtubule binding region (MTBR) within the tau protein is important in the pathogenic self‐association and aggregation of higher‐order tau species and critical for neuronal uptake of neurotoxic tau species via interactions with cell surface receptors. Increased MTBR tau concentrations in cerebrospinal fluid correlate with other markers of AD clinical progression.^1^

BMS‐986446 (formerly PRX005) is a humanized immunoglobulin class G1 kappa monoclonal antibody that binds to the MTBR of the tau protein and represents a novel potential therapeutic for early AD.

In vitro activity studies demonstrated that BMS‐986446 and its murine parent antibody 3D6 equivalently inhibit the uptake of aggregated tau into neurons, inhibit tau‐induced neurotoxicity, and promote uptake of aggregated tau into phagocytic cells preventing further cell‐to‐cell tau transmission.^2^

The TargetTau‐1 study (NCT06268886) will assess efficacy, safety, and tolerability of BMS‐986446 in patients with early AD.

**Methods:**

This phase 2, randomized, double‐blind, placebo‐controlled trial is enrolling adults aged 50 to 80 years with mild cognitive impairment or mild AD dementia (**Figure**). The 76‐week double‐blind treatment period is followed by a planned optional 96‐week open‐label extension. The primary endpoint is mean change from baseline in the Clinical Dementia Rating Sum of Boxes score at week 76. Primary and secondary endpoints are shown (**Figure)**.

**Results:**

The TargetTau‐1 study began enrollment on March 20, 2024, and aims to randomize 475 participants (4:3:3 to placebo, BMS‐986446 dose 1, or BMS‐986446 dose 2) across 14 countries in North America, Europe, and Asia‐Pacific regions.

**Conclusions:**

The phase 2 TargetTau‐1 trial will assess efficacy, safety, and tolerability of BMS‐986446, an anti‐MTBR tau monoclonal antibody, in patients with early AD.

**References**:

1. Horie K et al. *Nat Med* 2023;29:1954‐1963.

2. Nolan P et al. Poster presented at the ADII; June 9‐11, 2022; London, United Kingdom.